# Keratinases Produced by *Aspergillus stelliformis*, *Aspergillus sydowii,* and *Fusarium brachygibbosum* Isolated from Human Hair: Yield and Activity

**DOI:** 10.3390/jof7060471

**Published:** 2021-06-10

**Authors:** Suaad S. Alwakeel, Fuad Ameen, Hussah Al Gwaiz, Hana Sonbol, Salma Alghamdi, Ahmad M. Moharram, Osama A. Al-Bedak

**Affiliations:** 1 Department of Biology, College of Science, Princess Nourah bint Abdulrahman University, Riyadh 11671, Saudi Arabia; ssalwakeel@pnu.edu.sa (S.S.A.); hialgwaiz@pnu.edu.sa (H.A.G.); HSSonbol@pnu.edu.sa (H.S.); saAtAlghamdi@pnu.edu.sa (S.A.); 2 Department of Botany & Microbiology, College of Science, King Saud University, Riyadh 11451, Saudi Arabia; 3 Department of Botany and Microbiology, Faculty of Science, Assiut University, Assiut 71511, Egypt; ahmed.marzouk@science.au.edu.eg; 4 Assiut University Mycological Centre (AUMC), Assiut University, Assiut 71511, Egypt; osamaalbedak@gmail.com

**Keywords:** keratin, microbial keratinases, feather, waste management, enzymes, biotechnology

## Abstract

Twenty fungal strains belonging to 17 species and isolated from male scalp hair were tested for their capacity to hydrolyze keratinous material from chicken feather. The identification of the three most efficient species was confirmed by sequencing of the internal transcribed spacer (ITS) region of rDNA. Activities of fungal keratinases produced by *Aspergillus stelliformis* (strain AUMC 10920), *A. sydowii* (AUMC 10935), and *Fusarium brachygibbosum* (AUMC 10937) were 113, 120, and 130 IU mg^−1^ enzymes, respectively. The most favorable conditions were at pH 8.0 and 50 °C. Keratinase activity was markedly inhibited by EDTA and metal ions Ca^+2^, Co^+2^, Ni^+2^, Cu^+2^, Fe^+2^, Mg^+2^, and Zn^+2^, with differences between the fungal species. To the best of our knowledge, this is the first study on the activity of keratinase produced by *A. stelliformis*, *A. sydowii,* and *F. brachygibbosum*. *F. brachygibbosum* keratinase was the most active, but the species is not recommended because of its known phytopathogenicty. *Aspergillus sydowii* has many known biotechnological solutions and here we add another application of the species, as producer of keratinases. We introduce *A. stelliformis* as new producer of active fungal keratinases for biotechnological solutions, such as in the management of keratinous waste in poultry industry.

## 1. Introduction

Keratins are structural elements of wool, hooves, horns, hair, nails, and feathers. Forty million tons of keratinous waste is generated in the USA, Brazil, and China per year [1]. The meat industry—in slaughterhouses—generates millions of tons of keratinous waste in the world annually [2]. Chicken feathers alone are generated, up to two million tons [3]. In addition, the fur industry and barbershops generate keratinous waste. Only a small part of waste, mostly from slaughterhouses, is utilized as animal feed. Keratins are insoluble fibrillar proteins of the exterior defensive surfaces of vertebrates. Keratinous materials are known for their high stability owing to the firm stabilization of their polypeptide chains and the many disulfide bonds that cross these chains [4]. Keratins are not degraded by common protein breaking enzymes, i.e., proteases, such as pepsin and papain. Managing keratinous waste needs a low-cost solution, especially in developing countries.

Keratinous substrates are known to be degraded by fungi and bacteria that produce extracellular keratinolytic enzymes, i.e., keratinases [5,6,7]. Keratinophilic fungi are commonly found from many habitats. They have been found in soils, from Antarctica to the tropics, as well as from agricultural soils [8]. Keratinases are usually extracellular inducible enzymes secreted by different fungal genera, such as *Aspergillus*, *Chrysosporium*, *Trichophyton*, and *Microsporum* [9,10,11]. They are protease enzymes with widespread use in various industries. For instance, keratinase powder, produced using the bacterium *Bacillus licheniformis* and the fungus *Parengyodontium album*, is sold commercially. Keratinases are used in pharmaceutical industries, such as in vaccine production and preparation of bioactive peptides and serums. They are useful in the treatment of calluses, keratinized skin, psoriasis, and acne [12]. Manufacturing of cosmetic products, such as anti-dandruff shampoos, nutritional lotions, and creams use keratinases. They are commonly used in feed formulas, nitrogen fertilizers, and the leather industry [5,13,14,15]. As a novel solution, they may be used to treat wastewater containing keratin waste [16].

Keratinolytic fungi include Acremonium, Aphanoascus, Aspergillus, Chrysosporium, Cladosporium, Doratomyces, *Fusarium*, Lichtheimia, Microsporum, Paecilomyces, Scopulariopsis, Trichoderma, and Trichophyton. Many of these fungi are pathogenic. The pathogenicity and virulence of some fungi are often due to the high capability of the fungal strains to degrade both hard and soft types of keratin [17]. However, industrially interesting fungi are the non-pathogenic fungi that do not cause infections. Therefore, more research for suitable microorganisms is needed. Keratinases are active within a broad range of temperatures (40–70 °C) and pH values (6–11) [1,6,12,18,19,20,21,22]; thus, optimum conditions need to be studied for biotechnological solutions.

The aim of the study was to find suitable and effective fungal species able to degrade keratinous materials to be used in different biotechnological applications. We isolated fungi from human hair and, after a preliminary experiment, chose the best three species to measure the yield and the activity of keratinases the fungi produced.

## 2. Materials and Methods

### 2.1. Keratin Powder Preparation

Chicken feathers (50 g) collected from poultry farms located in the Assiut district, Egypt, were defatted with chloroform–methanol (1:1) in continuous agitation for 24 h, then washed three times with distilled water, and dried in the air. For keratin extraction, the chicken feathers were immersed in 1000 mL of 0.5 M sodium sulfide for 6 h with continuous agitation at ambient conditions. Soluble keratin was first centrifuged for 10 min at 10,000× *g* and then precipitated from the supernatant using 70% ammonium sulfate. The precipitate was washed four times with distilled water, dried at 40 °C, and used as chicken keratin powder in keratinase assay experiments.

### 2.2. Preliminary Experiment/Submerged Fermentation

Twenty fungal strains (17 species) previously [23] isolated from scalp hair samples of males in Riyadh city, Saudi Arabia, were used individually as three replicates. Fungi were preliminary identified morphologically and deposited in the Culture Collection of the Assiut University Mycological Centre (AUMC), with accession numbers in Table 1. Fungi were revived and tested for their purity and viability on Czapek’s agar medium (HiMedia, Mumbai, India).

Sterilized sucrose-free Czapek’s broth containing 5.0% (weight/weight) chicken feathers as the sole source of carbon and 0.1% glucose was used as a fermentation medium. In a 250 mL Erlenmeyer flask, 50 mL of fermentation medium was inoculated with 1 mL of cell suspension of the tested fungi. Spore suspension (1 mL) containing 1.8 × 10^8^ spore mL^−1^ from 7-day-old culture of each fungus was inoculated into their own fermentation flasks (100 mL broth) and incubated at 30 °C for 15 days under shaking at 150 rpm. Then, supernatants were obtained by centrifugation at 10,000× *g* for 10 min, and cell-free supernatants were used as the raw microbial keratinase enzymes in the preliminary keratinase activity assay.

### 2.3. Keratinase Activity Assay

The reaction mixture contained 1.0 mL of the cell-free supernatant + 0.01 g chicken keratin powder (prepared in 1 mL of 50 mmol citrate buffer pH 5.0). The mixture was incubated in a water bath at 50 °C for 60 min. The reaction was stopped by adding 2.0 mL 10% trichloroacetic acid (TCA). The resulting precipitate was separated by centrifugation at 10,000× *g* for 10 min. Then, 0.2 mL of the supernatant was diluted to 1.0 mL with purified water, and 5.0 mL of alkaline cupper reagent (sodium carbonate, 40 g; tartaric acid, 7.5 g; copper sulfate, 4.5 g and distilled. water, 1000 mL; final pH 9.9 ± 0.5) was added. Afterwards, 0.5 mL of the Folin–Ciocalteu reagent was added and the tubes were kept in the dark for 30 min to allow the blue color formation. Negative control was prepared by incubating the enzyme solution with 2 mL of 10% TCA without keratin. Absorbance was measured at 660 nm (UV-visible spectrophotometer; T80+; UK), using tyrosine as the standard. One unit of keratinolytic activity corresponds to the enzyme amount that releases 1 μmol tyrosine mL^−1^ min^−1^ under standard test conditions [24], according to the L-tyrosine standard curve (Equations (1)–(3)) [24].

Concentration of L-tyrosine = absorbance/(0.0018 × 1000) mg/mL (=g/L)(1)

Keratinase activity = (concentration of L-tyrosine)/0.0001812 IU/mL/min(2)

Keratinase activity can be also expressed as IU/g (= IU/mL/min × 100).(3)

Total protein content was measured with the method of [25], using bovine serum albumin (BSA) as the standard and the specific keratinase activity per mg protein was calculated.

### 2.4. Molecular Identification of the Potent Strains

The three most active strains in the preliminary experiment were identified by sequencing. For the DNA extraction, small pieces of fungal mycelia from 7-day-old cultures of *Aspergillus* strains (AUMC 10920 and AUMC 10935) grown on malt extract agar (MEA) and *Fusarium* strain (AUMC 10937) on potato dextrose agar (PDA) at 25 °C were transferred individually to 2 mL Eppendorf tubes. DNA extraction was performed as described by Moubasher et al. [26]. The PCR reaction was carried out in SolGent company (South Korea) using the universal primers ITS1 (5TCC GTA GGT GAA CCT GCG G 3), and ITS4 (5TCC TCC GCT TAT TGA TAT GC 3), in the reaction mixture described by [27,28,29]. Sequences obtained from SolGent Company were compared to sequences from GenBank using MAFFT (version 6.861b) with the default options [30]. Alignment gaps and parsimony uninformative characters were chosen as described by Criscuolo and Gribaldo [31]. Maximum likelihood (ML) and maximum parsimony (MP) phylogenetic analyses were conducted using PhyML 3.0 [32]. The robustness of the most parsimonious trees was tested by 100 bootstrap replications [33]. The best optimal model of nucleotide replacement for ML analyses was calculated using Smart Model Selection (SMS) version 1.8.1 [34]. The phylogenetic tree was prepared with FigTree version 1.4.3 [35] and edited with Microsoft Power Point (2016).

### 2.5. Experiment to Produce Keratinases Using the Three Most Active Strains

The three most active fungal strains were used in the submerged fermentation incubation, carried out as described for the preliminary submerged fermentation incubation above. Erlenmeyer flasks (500 mL) containing 100 mL of fermentation medium were used. After the fermentation period, the cell-free supernatants were subjected to 60% ammonium sulfate precipitation. The precipitated proteins were isolated and lyophilized using a freeze dryer (VirTis, model #6KBTES-55, NY, USA). Lyophilized keratinases were dissolved separately in citrate buffer (pH 5.0) and dialyzed twice at room temperature against the same buffer for 2 h, removing the buffer every time. Then, they were stored overnight at 4 °C to exclude small molecules. The dialyzed keratinases were then lyophilized using a freeze dryer, weighed, and used as partially purified fungal keratinase enzymes to measure the activity of the enzymes and their optimal conditions.

### 2.6. Effect of PH and Temperature on the Activity of Partially Purified Keratinases

The partially purified fungal keratinases of the three selected fungi were used in the reaction mixture that contained 0.01 g keratinase and 0.01 g chicken keratin powder (each was prepared independently in a 50 mmol citrate buffer solution of 1.0 mL). The different experiments were prepared as three replicates and the absorbances were measured as above. Keratinase activity was calculated and expressed as kilo unit per g keratinase (KU g^−1^ keratinase).

Eight pH values (3.0–10.0) were tested. The buffers used were citrate buffer (pH 3.0–6.0), phosphate buffer (pH 7.0–8.0), and borate buffer (pH 9.0–10.0). Then, six temperatures (30–80 °C) at the optimum pH value of each microbial keratinase was tested. Divalent metal ions (Ca^+2^, Co^+2^, Ni^+2^, Cu^+2^, Fe^+2^, Mg^+2^, and Zn^+2^) were tested by adding at the concentration of 5 mmol mL^−1^ as CaCl_2_, CoCl_2_, NiCl_2_, CuSO_4_, FeSO_4_, MgSO_4_, and ZnSO_4_. An enzyme inhibitor was tested using 5 mmol mL^−1^ ethylenediaminetetraacetic acid (EDTA). The activity of the microbial keratinase in the absence of metal ions or EDTA were measured under standard conditions to define 100% activity in optimal conditions.

## 3. Results

### 3.1. Preliminary Experiment

Six strains exhibited keratinase activity above 1500 IU mL^−1^ min^−1^ (Table 1). *Fusarium brachygibbosum* was the most active (3554 IU mL^−1^ min^−1^) followed by *A. sydowii* and *Aspergillus stelliformis* with the activities of 3336 and 3523 IU mL^−1^ min^−1^, respectively. Keratinase activity values from *Aspergillus ustus*, *Alternaria alternata* (AUMC 10932), and *Chaetomium globosum* showed 2091, 1681, and 1600 IU mL^−1^ min^−1^, respectively. The remaining tested fungi showed low keratinase activity ranging from 52 to 1398 IU mL^−1^ min^−1^.

### 3.2. Fungi Producing Active Microbial Keratinases

**Figure 1:**
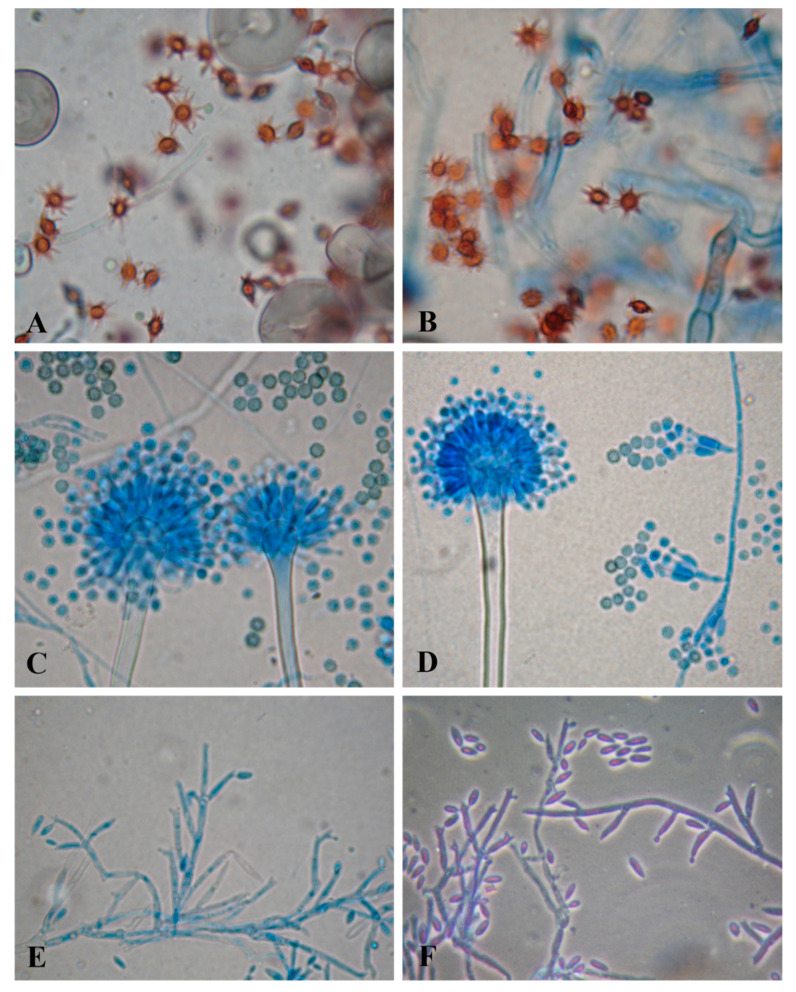
Light brown spores of *A. stelliformis* (**A**,**B**), biseriate conidiophores, and spinulose conidia of *A. sydowii* (**C**,**D**), and relatively curved and fusiform microconidia of *F. brachygibbosum* originated from mono and polyphialides (**E**,**F**).

**Figure 2:**
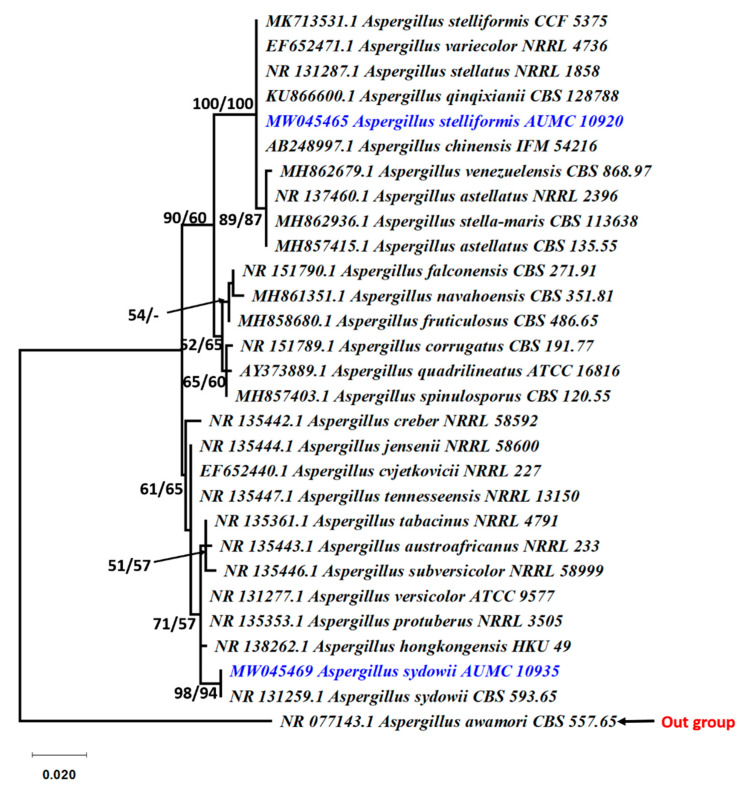
Phylogenetic tree generated from MP analysis based on ITS sequence data of *Aspergillus stelliformis* AUMC 10920 and *A. sydowii* AUMC 10935 associated to other related genes in Aspergillaceae. Blue color refers to the species in this study. Bootstrap support values (100 replications) for ML/MP combination equal to or greater than 50% are indicated at the respective nodes. The tree is rooted to *Aspergillus awamori*-CBS 557.65 as the out group.

**Figure 3:**
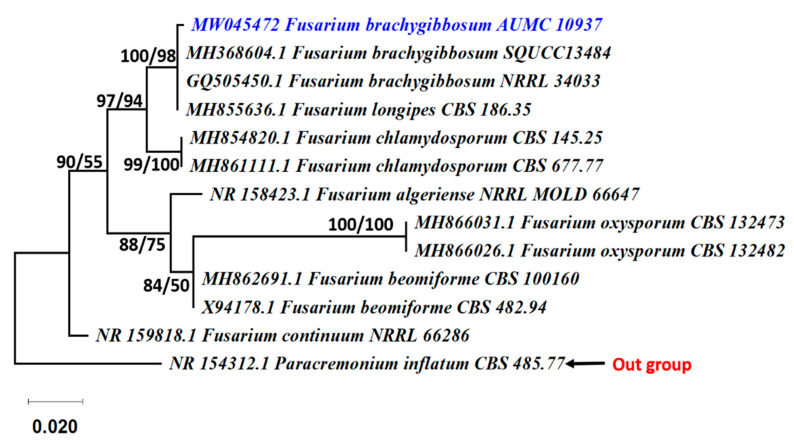
Phylogenetic tree generated from MP analysis based on ITS sequence data of *F. brachygibbosum* AUMC 10937 associated to other related genes in Nectriaceae. Blue color refers to the species in this study. Bootstrap support values (100 replications) for ML/MP combination equal to or greater than 50% are indicated at the respective nodes. The tree is rooted to *Paracremonium inflatum* CBS 485.77 as the out group.

The three most active fungal species were confirmed by by phylogenetic analysis as *Aspergillus stelliformis*, *A. sydowii,* and *Fusarium brachygibbosum* (Figure 1). The phylogenetic tree showed the relationship of our *Aspergillus* strains AUMC 10920 and AUMC 10935 to the other *Aspergillus* species. The *Aspergillus* species in this analysis showed 100% similarity to *A. stelliformis* CCF 5375 and *A. sydowii* CBS 593.65 (Figure 2). Sequencing data were submitted to GenBank and assigned accession numbers as MW045465 for *A. stelliformis* and MW045469 for *A. sydowii*. It is worth it to mention that *A. stelliformis* is a newly recorded species related to *Aspergillus* section Nidulantes that accommodates *A. nidulans* and other species developing biseriate conidiophores with light brown-pigmented stipes, and, if present, the ascomata embedded in masses of Hülle cells. The *Fusarium* strain AUMC 10937 was identified as *F. brachygibbosum* with GenBank accession number of MW045472 (Figure 3).

### 3.3. Yield and Activity of Keratinases

**Figure 4:**
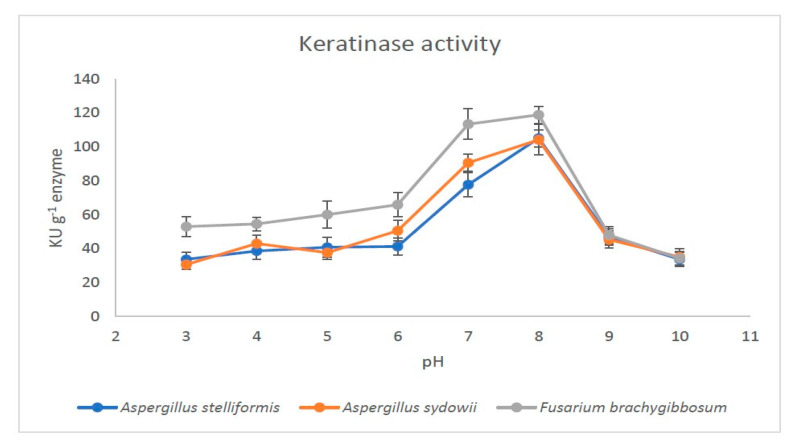
Effect of pH on the activity of fungal keratinases produced by *A. stelliformis, A. sydowii,* and *F. brachygibbosum*.

In submerged fermentation, the three fungi produced keratinases with relatively high yield. It was possible to produce 4.0 g keratinase powder from *A. stelliformis*, 4.5 g from *A. sydowii*, and 3.7 g from *F. brachygibbosum* per liter of fermentation medium. The keratinases appeared to be active; the highest activity was reached at pH 8.0 for each microbial keratinase, and were 105, 104, and 119 KU/g keratinase for *A. stelliformis*, *A. sydowii*, and *F. brachygibbosum*, respectively (Figure 4). The specific activities were 4223, 3522, and 3277 IU/mg protein, respectively.

**Figure 5:**
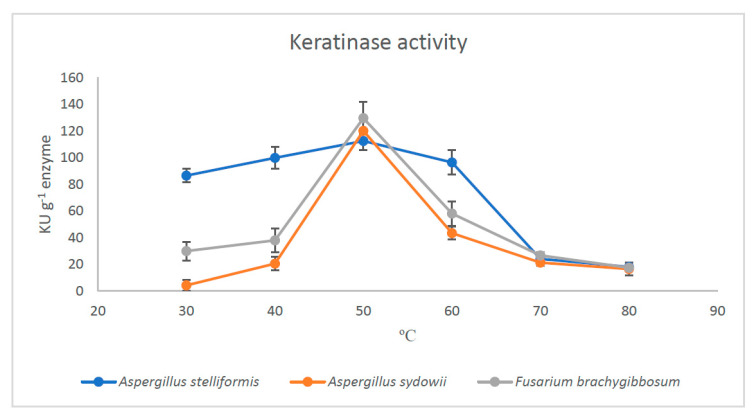
The Effect of temperature on the activity of keratinases produced by *A. stelliformis, A. sydowii,* and *F. brachygibbosum*.

The optimum temperature was 50 °C (pH 8.0) for each of the three microbial keratinases. The activity values increased to 113, 120, and 130 KU/g keratinase produced by *A. stelliformis*, *A. sydowii,* and *F. brachygibbosum*, respectively (Figure 5). A notable observation was that *F. brachygibbosum* was active at a wide temperature range (30–60 °C). The optimum temperature (50 °C) also increased the specific activity of the three keratinases to 4521, 4060, and 3573 IU/mg protein for *A. stelliformis*, *A. sydowii,* and *F. brachygibbosum*, respectively.

EDTA and the metal ions had strong inhibitory effects on the activity of the keratinases produced when tested under the optimum conditions observed (pH 8 and 50 °C). The maximum inhibitory effect was with EDTA in case of the keratinases produced *by A. stelliformis* and *A. sydowii*, but not by *F. brachygibbosum* (Table 2). For *F. brachygibbosum,* the inhibitory metal ions were Ca, Cu, and Zn.

## 4. Discussion

Microbial keratinases are considered highly useful in many biotechnological applications. For instance, they are known as plant growth promoters [36]. A more recent application involves treating keratinous waste produced in agriculture and leather industries. This waste is difficult to treat with proteases, such as papain, pepsin, and trypsin [30]. Finding efficient degraders of keratin offers possibilities to treat waste from cattle, poultry, and leather industries, and keratinases has been studied with the aim to degrade the different waste material [37,38]. The advantages of using natural keratinophilic microbes in producing enzymes are the low cost and that the byproducts are nontoxic (and can even be utilized elsewhere) [39]. Byproducts, such as amino acids, polypeptides, vitamins, and detergent additives are promising novel applications that improve the sustainability of agriculture [40,41]. The mechanisms behind the degradation of keratinous materials still requires further studies. The process of keratinolysis can be catalyzed by a single keratinase, or more efficiently, in synergy with other enzymes [7]. Other enzymes such as disulfide reductases catalyze the breakage of disulfide bonds. Metabolic cooperation with amino acid metabolism, urea cycle, and disulfide reduction was revealed using metagenomic analysis [42]. Different reducing agents, such as β-mercaptoethanol and dithiothreitol were present when keratinases produced by fungi and actinomycetes. *Thermoactinomyces* sp., *Trichophyton* sp., *Streptomyces* sp., *A. parasiticus* and *A. niger* were compared in a review of Peng et al. [43].The mechanism behind the keratin degradation has been studied with keratinases produced by *Bacillus thuringiensis* isolated from donkey hairs. Scanning electron microscopy and Fourier transform infrared spectrophotometry showed the disintegration and disruption of the disulphide bonds of the keratin structure [44]. The use of natural microbes reduces the costs of the enzyme production and, at the same time, offers economic processes to waste management [2]. Each of the three species studied appeared to be good candidates to produce active keratinases. Different species of *Aspergillus* have been reported often as potential producers of keratinases the optimum conditions varying a lot. The optimum conditions for *Aspergillus terreus* in a 25-days incubation were 40 °C and pH 8 [45]. The mutants of 28 strains of *A. niger* produced varying amounts of keratinases on solid state fermentation in basal medium containing chicken feathers after 7 d; the highest activity of keratinases was achieved at pH 5 [46]. When cultivated in feather meal basal medium containing 2% (*w*/*v*) chicken feather for 16 days, the optimum of *A. flavus* was at pH 8 and 28 °C [47]. *Aspergillus* sp. DHE7 recovered from poultry farm soil in Egypt had the maximum keratinase activity 199 IU/mL in a 2 % chicken feather substrate when incubated for 4 days at 30 °C and pH 6.0 [48]. The addition of 0.5 % sucrose as a supportive carbon source raised the keratinase activity to 226 IU/mL. The best substrates for the keratinase activity were goat hair (452 IU/mL), turkey feathers (435 IU/mL) and sheep wool (322 IU/mL). Our *Aspergillus* species had a clearly higher temperature optimum, 50 °C.

In addition to *Aspergillus*, several other fungal genera have been shown to produce active keratinases as well. *Scopulariopsis brevicaulis* keratinolytic activity was the highest on chicken feathers followed by human nails and human hair [49]. *Cochliobolus hawaiiensis* achieved the maximum development of alkaline keratinase after an incubation for 15 d at 30 °C and pH 9.5 [50]. *Chrysosporium tropicum* optimum production of keratinase in medium containing chicken feathers was after 21 d at 25 °C using 1% glucose as carbon source [39]. *Trichophyton ajelloi* had its maximum enzyme activity (6.3 KU/mL) at 30 °C [51]. Keratinolytic activity of *Chrysosporium tropicum* was the highest (8.6 KU/mL) on the 40th day of the incubation [40]. For *Microsporum gypseum* (78 KU/mL) and *M. canis* (76 KU/mL) the highest activity was recorded on the 20th day of the incubation [41]. *Bacillus thuringiensis* showed the activity of 422 U/mL at 50 °C and pH 9 [44]. In general, it is difficult to compare the values of enzyme activity between different studies due to slight differences in methodology. Therefore, the comparisons should be done with caution.

Many studies have confirmed the dependence of microbial keratinase activity from metals [52,53]. This was the case with our three species *A. sydowii, A. stelliformis and F. brachygibbosum* as well. We found no previous information about our species but a feather-degrading culture of *Aspergillus oryzae* was activated by Ca and Ba ions while inhibited by EDTA and Pb ions [54]. Under solid state fermentation with chicken feathers, *A. flavipes* keratinase activity was greatly inhibited by EDTA, Hg^2+^, Fe^3+^ [55]. No great effect on *A. flavipes* keratinase was observed due to the presence of Zn^2+^, Mg^2+^ and Cu^2+^. The published research reveals that different fungal species have highly variable optimum conditions. This indicates that the optimum culturing conditions and the inhibitory compounds must be examined for each species separately.

Many of the *Fusarium* species are known as opportunistic pathogens causing many plant diseases, among them is one of the most destructive plant diseases *Fusarium* wilt of banana [56]. *Fusarium brachygibbosum* specifically has caused, for instance, leaf spot disease of date palms and dry rot disease of citrus trees [57,58]. It was reported as a causative agent of date palm wilting disease although its pathogenicity was assessed as low [59]. *Fusarium* species are known as toxigenic fungi secreting mycotoxins in food and feed such as cereals [60,61]. Although *F. brachygibbosum* keratinase was the most active in a wide temperature range tested in our study, it must be recommended with caution because of its known pathogenicity to plants. 

*Aspergillus sydowii* is not known as especially pathogenic but its role as an opportunistic pathogen causing diseases for instance in coral reefs has been studied in several seas [62,63]. *Aspergillus sydowii* is known as a species tolerating highly saline conditions and reported to have potential to be used in different biotechnological solutions. Several *Aspergillus* species in general are known to have adverse health effects on humans [64]. Most often, the fungi have been reported to cause local infections and allergy [65]. However, we found no reports about severe health effects of either of our *Aspergillus* species. Its potential has been verified for instance, in the remediation of polyaromatic hydrocarbons, pesticides, and pharmaceutical compounds [66,67]. *Aspergillus sydowii* was observed to adsorb heavy metals (Cd) and degrade pesticides (trichlorfon) in vitro [68]. In addition, *A. sydowii*, as a producer of anthocyanins, has many potential applications in human health and for instance as natural dyes of foodstuff [69].

*Aspergillus sydowiii* has been shown to produce many different enzymes that have potential in many biotechnological applications. Cellulase was produced under submerged fermentation [70] and xylanases under solid-state fermentation [71]. Moreover, tannases [72] and lignocellulosic enzymes offer possibilities to food and bioenergy applications [73]. We can add keratinases the list of *A. sydowiii* for the first time.

Only some information about *A. stelliformis* was found [74]. No indication about its bioactivities nor mentions about its potential use in biotechnology were found. We report, for the first time, the potential of *A. stelliformis* to produce keratinases and its potential to be used in applications to degrade keratinous material.

## 5. Conclusions

*Aspergillusstelliformis*, *A. sydowii,* and *F. brachygibbosum* appeared to produce keratinases that had high activity. *F. brachygibbosum* keratinase was the most active in a wide temperature range. However, as a producer of keratinases, it must be used with caution and cannot be recommended because of its known phytopathogenicity. *Aspergillus sydowii* is known as a species with several potential biotechnological solutions. To this long list published using *A. sydowii*, we can add the production of the active keratinase enzyme. We also introduce a new-recorded species, *A. stelliformis*, to be used in biotechnological solutions as a producer of active microbial keratinase. Although further studies are required, both *Aspergillus* species could be used in degrading problematic and recalcitrant keratinous waste and in developing sustainable agriculture.

## Figures and Tables

**Table 1:** Activity of fungal keratinases (mean ± SD, n = 3). AUMC no. = Culture Collection of the Assiut University Mycological Centre accession numbers.

Fungal Species	AUMC No.	Relative Activity IU mL^−1^ min^−1^
*Alternaria alternata* (Fries) Keissler	10926	689 ± 42
*Alternaria alternata* (Fries) Keissler	10932	1680 ± 120
*Alternaria botrytis* Woudenberg and Crous	10931	1211 ± 98
*Alternaria botrytis* Woudenberg and Crous	10936	1018 ± 87
*Alternaria chlamydosporigena* Woudenberg and Crous	10915	1398 ± 154
*Aspergillus nidulans* Winter	10933	935 ± 70
*Aspergillus niger* van Tieghem	10912	52 ± 23
*Aspergillus stelliformis* F. Sklenar, Jurjević and Hubka	10920	3336 ± 169
*Aspergillus sydowii* (Bainier and Sartory) Thom and Church	10935	3523 ± 188
*Aspergillus ustus* (Bainier) Thom and Church	10934	2091 ± 197
*Aureobasidium pullulans* (de Bary) Arnaud	10914	129 ± 11
*Chaetomium globosum* Kunze	10941	1600 ± 156
*Curvularia tsudae* (Tsuda & Ueyama) Deng, Tan and Shivas	10940	570 ± 85
*Fusarium brachygibbosum* Padwick	10937	3554 ± 189
*Nodulisporium* sp.	10916	270 ± 20
*Penicillium chrysogenum* Thom	10913	589 ± 11
*Penicillium glabrum* (Wehmer) Westling	10929	218 ± 49
*Phoma herbarum* Westend	10919	258 ± 12
*Pyrenophora dematioidea* (Bubák & Wróbl.) Rossman and K.D. Hyde	10930	580 ± 33
*Pyrenophora dematioidea* (Bubák & Wróbl.) Rossman and K.D. Hyde	10938	512 ± 15

**Table 2:** Effect of metal ions and EDTA (5 mmol mL^−1^) on keratinase activity produced by different fungi (mean ± SD, n = 3). The results are expressed as the proportion of the activity in the tested inhibitory conditions, from the keratinase activity in the control without inhibitors.

Control Activity KUg^−1^ Enzyme	113	121	130
Inhibitor	*A. stelliformis*	*A. sydowii*	*F. brachygibbosum*
FeSO_4_	28 ± 4	27 ± 2	24 ± 3
ZnSO_4_	29 ± 3	18 ± 2	23 ± 4
CaCl_2_	34 ± 6	22 ± 6	22 ± 4
CuSO_4_	28 ± 3	22 ± 5	23 ± 2
NiCl_2_	30 ± 4	58 ± 9	45 ± 4
CoCl_2_	76 ± 8	33 ± 8	27 ± 4
MgSO_4_	27 ± 5	17 ± 5	53 ± 8
EDTA	7 ± 1	17 ± 4	52 ± 6

## Data Availability

All data related to this manuscript is incorporated in the manuscript only.
